# Semi-Preparative Separation of 10 Caffeoylquinic Acid Derivatives Using High Speed Counter-Current Chromatogaphy Combined with Semi-Preparative HPLC from the Roots of Burdock (*Arctium lappa* L.)

**DOI:** 10.3390/molecules23020429

**Published:** 2018-02-15

**Authors:** Zhenjia Zheng, Xiao Wang, Pengli Liu, Meng Li, Hongjing Dong, Xuguang Qiao

**Affiliations:** 1 College of Food Science and Engineering, Shandong Agricultural University, Taian 271018, China; pengyou-jia@163.com (Z.Z.); milly.liu@163.com (P.L.); Lemon982436@163.com (M.L.); 2 Key Laboratory of TCM Quality Control Technology, Shandong Analysis and Test Center, Qilu University of Technology (Shandong Academy of Sciences), Jinan 250014, China; wangx@sdas.org

**Keywords:** burdock root, new caffeoylquinic acid derivatives, high speed counter-current chromatography, semi-preparative HPLC

## Abstract

Burdock roots are healthy dietary supplements and a kind of famous traditional Chinese medicine, which contains large amounts of caffeoylquinic acid derivatives. However, little research has been reported on the preparative separation of these compounds from burdock roots. In the present study, a combinative method of HSCCC and semi-preparative HPLC was developed for the semi-preparative separation of caffeoylquinic acid derivatives from the burdock roots. The ethyl acetate extract of burdock roots was first fractionated by MCI macroporous resin chromatography and give three fractions (Fr. 1–3) from the elution of 40% methanol. Then, these three fractions (120 mg) were separately subjected to HSCCC for purification with the solvent system composed of petroleum ether-ethyl acetate-methanol-water at different volume ratios, and the mixtures were further purified by semi-preparative HPLC. As a result, a total of eight known caffeoylquinic acid derivatives including 3-*O*-caffeoylquinic acid (32.7 mg, 95.7%), 1,5-*O*- dicaffeoylquinic acid (4.3 mg, 97.2%), 3-*O*-caffeoylquinic acid methyl ester (12.1 mg, 93.2%), 1,3-*O*-dicaffeoylquinic acid (42.9 mg, 91.1%), 1,5-*O*-dicaffeoyl-3-*O*-(4-maloyl)-quinic acid (4.3 mg, 84.5%), 4,5-*O*-dicaffeoylquinic acid (5.3 mg, 95.5%), 1,5-*O*-dicaffeoyl-3-*O*-succinylquinic acid (8.7 mg, 93.4%), and 1,5-*O*-dicaffeoyl-4-*O*-succinylquinic acid (1.7 mg, 91.8%), and two new compounds were obtained. The new compounds were 1,4-*O*-dicaffeoyl-3-succinyl methyl ester quinic acid (14.6 mg, 96.1%) and 1,5-*O*-dicaffeoyl-3-*O*-succinyl methyl ester quinic acid (3.1 mg, 92.6%), respectively. The research indicated that the combination of HSCCC and semi-preparative HPLC is a highly efficient approach for preparative separation of the instability and bioactive caffeoylquinic acid derivatives from natural products.

## 1. Introduction

Burdock (*Arctium lappa* L.) is a biennial plant of the Asteraceae family and its root has been consumed as a tonic vegetable in Asia. Burdock root is not only a healthy and nutritive dietary supplement, it was also a folk herbal medicine with heat-clearing and detoxifying effect [1]. Pharmacological studies indicated that burdock root has antioxidant [2], antiallergic [3], anti-inflammatory [4] and hepatoprotective activities [5]. Caffeoyl quinic acid derivatives are considered to be one of the main active ingredients, such as 1,3-, 1,4-, 1,5-dicaffeoylquinic acids, which were all positional isomers in chemical structure [6,7]. 

Due to the similarity of caffeoylquinic acids, the purification was usually achieved by repeated reverse silica gel column chromatography in the past [8,9]. However, a crude sample is not acceptable when using expensive reverse silica gel, and it usually requires multi-steps of traditional chromatography for the pre-treatment of crude samples. These procedures have the drawbacks of long processing time and low repeatability. Moreover, some compounds with less stability will degrade during the repeat and long separation procedure. Therefore, it is necessary to develop an efficient and repeatable method for the rapid purification of caffeoylquinic acids from natural materials.

High-speed counter-current chromatography (HSCCC) is a continuous liquid-liquid partition chromatography based on partition of compounds between two immiscible liquid phases. It has many advantages such as no irreversible adsorption, low risk of sample denaturation, total sample recovery, and low cost [10,11]. This method has been successfully applied to fractionate and purify 3-caffeoylquinic acid and 3,5-, 4,5-dicaffeoylquinic acids from complex natural extracts with high efficiency [12,13,14]. However, there has been little research until now involving the purification of 1,3, 1,4-, 1,5-caffeoylquinic acids by HSCCC [15].

**Figure 1:**
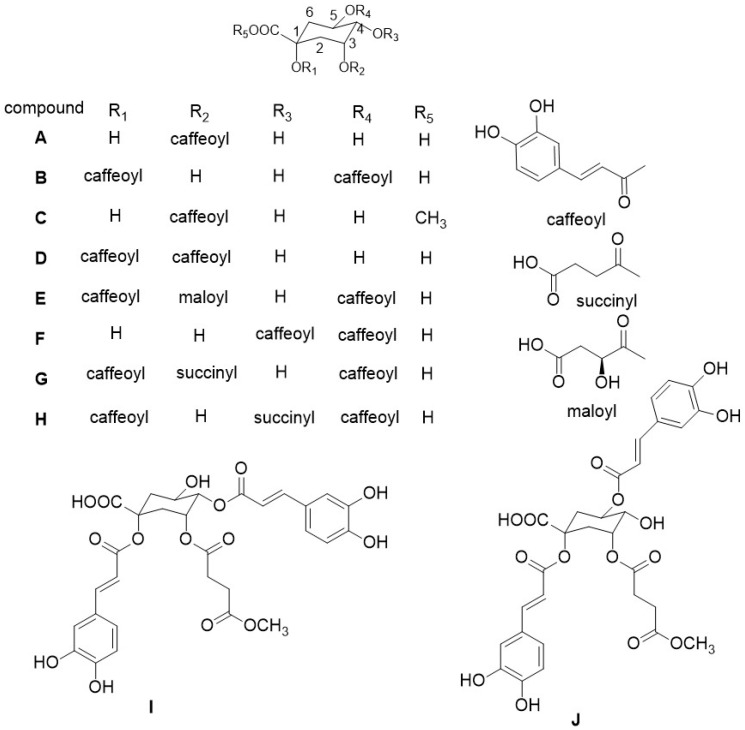
Chemical structures of ten caffeoylquinic acid derivatives from burdock roots.

In this work, the crude extract of burdock roots was fractionated into three fractions by MCI macroporous resin. HSCCC was developed for the purification and separation of caffeoylquinic acid derivatives from these three fractions, and the compounds with low purity were further purified by semi-preparative HPLC. Finally, 10 isomers of caffeoyl quinic acid derivatives were obtained, among which two were new (compounds **I**, **J**). The structure of these compounds is shown in Figure 1. 

## 2. Results and Discussion

### 2.1. Optimization of HSCCC Conditions

The search of appropriate solvent system plays an important role in separation of HSCCC. Suitable two-phase solvent system should satisfy several principles, such as the good solubility, high retention of the stationary phase, and the most important was the suitable partition coefficients (*K*_D_) for target compounds and separation factors between adjacent peaks [10,16]. To achieve a successful separation using HSCCC, according to the purification of 3,5-, 4,5-dicaffeoylquinic acids using HSCCC by wang et al. [14], and polarity, solubility of caffeoylquinic acid derivatives, the *K*_D_ values of these 10 compounds in the solvent system composed of Pet-EtOAc-MeOH-water were measured and are given in Table 1.

#### 2.1.1. Separation of Fr. 1 by HSCCC

**Figure 2:**
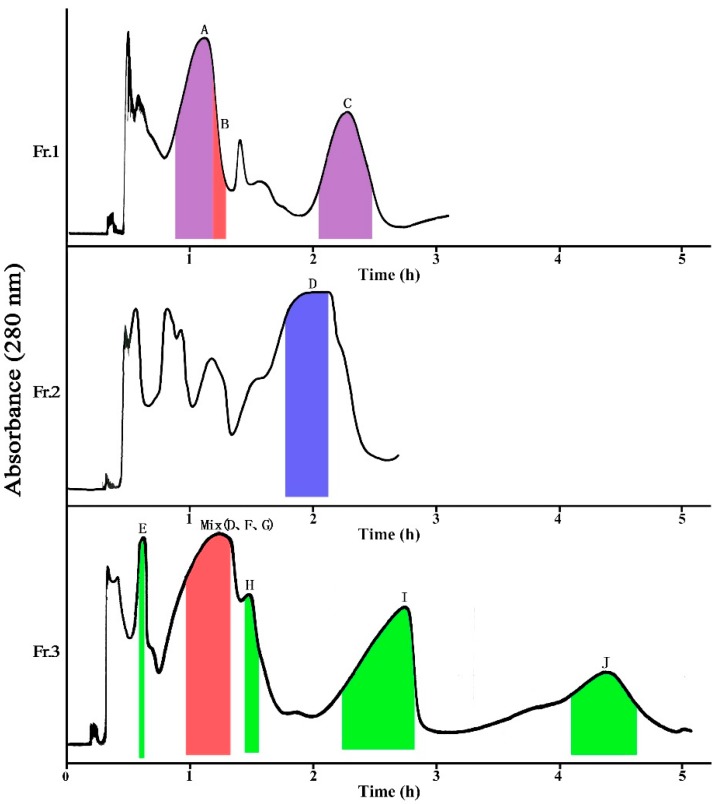
High-speed counter-current chromatography (HSCCC) chromatograms of Fr. 1–3.

As shown in Table 1, the solvent system composed of EtOAc–MeOH-water (5:0.5:5, *v*/*v*) provided suitable *K*_D_ value for compound **C** (2.21). However, the *K*_D_ values for compounds **A** and **B** were close to each other, which may result in the partially overlap of compounds **A** and **B** in HSCCC separation. In order to achieve good separation of compounds **A** and **B**, methanol was removed to reduce the elution ability of the mobile phase and improve the resolution, and the *K*_D_ values of compounds **A**–**C** were measured in the solvent system composed of EtOAc-water (5:5, *v*/*v*). From Table 1, it can be seen that EtOAc-water (5:5, *v*/*v*) still provided close *K*_D_ values for compounds **A** and **B** (0.55, 0.58) and larger *K*_D_ values for compound **C** (4.82), and thus the separation of compounds **A** and **B** may be very difficult using this solvent system. Considering the suitable *K*_D_ values and good separation factor of compound **C** in the solvent system composed of EtOAc-MeOH-water (5:0.5:5, *v*/*v*), the solvent system was used for the separation of Fr. 1 (Figure 2). When the two-phase solvent system was applied, compounds **A** (28.5 mg, peak **A**) and **C** (12.1 mg, peak **C**) were obtained with purities of 95.7% and 93.2%, respectively, from 120 mg of Fr.1 (Figure S2), and compound **B** was eluted together as a mixture (15.6 mg) with small portion of compound **A** after analysis by HPLC. 

The mixture (15.6 mg) was further purified by semi-preparative HPLC with the solvent of acetonitrile-water containing 0.1% HCOOH (25:75, *v*/*v*). Finally, compounds **A** and **B** can be largely isolated from the mixture, and obtained 4.2 mg of compound **A** and 4.3 mg of compound **B** with purities of 97.9% and 97.2% (Figure S2), respectively.

#### 2.1.2. Separation of Fr. 2 by HSCCC

According to the separation of Fr. 1 by HSCCC and polarity of compound **D** in Fr. 2, the *K*_D_ values of compound **D** in the solvent system composed of EtOAc-MeOH-water (5:1:5, *v*/*v*) was measured, and the *K*_D_ value (26.88) was too large that it will consume a large amount of solvent to elute compound **D**. By adding a small amount of Pet and increasing the ratio of MeOH, the *K*_D_ values changed to be smaller (Table 1). The solvent system composed of Pet-EtOAc-MeOH-water (1:4:1:4, *v*/*v*) provided suitable *K*_D_ values for compound **D** (2.03). When this solvent system was used for the purification (Figure 2), 42.9 mg of compound **D** was obtained with purity of 91.1% from 120 mg of Fr. 2 (Figure S3). 

#### 2.1.3. Separation of Fr. 3 by HSCCC

As shown in Table 1, the solvent system composed of Pet-EtOAc-MeOH-water (1:4:1:4, *v*/*v*) provided suitable *K*_D_ values for compounds **E**–**I** (0.50, 2.10, 2.21, 3.47, 7.88). However, the *K*_D_ values of compounds F and G were very close (2.10, 2.21), and the two compounds would be eluted together in the HSCCC separation. Figure 2 shows the HSCCC chromatography for the separation of Fr. 3 using the solvent system composed of Pet-EtOH-MeOH-water (1:4:1:4, *v*/*v*), and five peaks were obtained. After analysis by HPLC, peaks **E**, **H**–**J** all contain only one compounds. Though the compounds of peak **I** and **J** have the same elution time in HPLC, they were two different compounds, which were named compound **I** and **J**, separately. After being collected and dried, 4.3 mg of compound **E**, 1.7 mg of compound **H**, 14.6 mg of compound **I**, and 3.1 mg of compound **J** were obtained from 120 mg of Fr. 3. The purities of these compounds were all determined by peak area normalization method at 280 nm, and they were 84.5%, 91.8%, 96.1%, and 92.6% (Figure S4), respectively. 

Compounds **F** and **G** were eluted as a mixture. The mixture was further purified by semi-preparative HPLC with methanol-water containing 0.1% HCOOH (25:75, *v*/*v*). As a result, 5.3 mg of compound **F** and 8.7 mg of compound **G** were obtained from 39.8 mg of mixture with purity of 95.5% and 93.4% (Figure S4), respectively. 

In all, six purified compounds were obtained after one-step separation of HSCCC and semi-preparative HPLC separation from Fr. 3.

Fr. 1 Solvent systems: Pet-EtOAc-MeOH-H_2_O (0:5:0.5:5, *v*/*v*); Fr. 2 Solvent systems: Pet-EtOAc-MeOH-H_2_O (1:4:1:4, *v*/*v*); Fr. 3 solvent system: Pet-EtOAc-MeOH-H_2_O (1:4:1:4, *v*/*v*). sample size: 120 mg; flow-rate: 5.0 mL/min; detection: 280 nm. 

### 2.2. Identification of Compounds

#### 2.2.1. Identification of Known Compounds

Compound **A** ESI-MS, *m/z* 355.1035 [M + H]^+^, 353.0890 [M − H]^−^; ^1^H-NMR (DMSO-*d*_6_, 400 MHz) *δ* (ppm): 1.76–2.03 (4H, m, H-2, 6), 5.08 (1H, m, H-3), 3.58 (1H, m, H-4), 3.93 (1H, m, H-5), 7.04 (1H, br s, H-2′), 6.77 (1H, d, *J* = 8.0 Hz, H-5′), 6.98 (1H, d, *J* = 7.8 Hz, H-6′), 7.42 (1H, d, *J* = 16.0 Hz, H-7′), 6.15 (1H, d, *J* = 16.0 Hz, H-8′); ^13^C-NMR (DMSO-*d*_6_, 100 MHz) *δ* (ppm): 73.6 (C-1), 37.2 (C-2), 68.3 (C-3), 70.5 (C-4), 70.9 (C-5), 37.2 (C-6), 175.0 (C-7), 125.6 (C-1′), 114.8 (C-2′), 145.6 (C-3′), 148.3 (C-4′), 115.8 (C-5′), 121.4 (C-6′), 144.9 (C-7′), 114.3 (C-8′), 165.8 (C-9′). Compared with reported data [17], compound **A** was identified as 3-*O*-caffeoylquinic acid. 

Compound **B** ESI-MS, *m/z* 517.1313 [M + H]^+^, 515.1202 [M − H]^−^; ^1^H-NMR (DMSO-*d*_6_, 400 MHz) *δ* (ppm): 1.76–2.47 (4H, m, H-2, 6), 3.98 (1H, m, H-3), 3.50 (1H, m, H-4), 5.28 (1H, m, H-5), 7.01, 6.91 (each 1H, br s, H-2′, 2″), 6.67, 6.53 (each 1H, d, *J* = 8.0 Hz, H-5′, 5″), 6.87, 6.63 (each 1H, d, *J* = 8.0 Hz, H-6′, 6″), 7.42, 7.40 (each 1H, d, *J* =16.0 Hz, H-7′, 7″), 6.20, 6.07 (each 1H, d, *J* = 16.0 Hz, H-8′, 8″); ^13^C-NMR (DMSO-*d*_6_, 100 MHz) *δ* (ppm): 80.1 (C-1), 34.2 (C-2), 66.5 (C-3), 73.2 (C-4), 71.4 (C-5), 39.4 (C-6), 173.1 (C-7), 125.7, 125.8 (C-1′, 1″), 116.2, 115.7 (C-2′, 2″), 145.3, 146.0 (C-3′, 3″), 148.6, 148.9 (C-4′, 4″), 116.4, 116.3 (C-5′, 5″), 120.5, 121.4 (C-6′, 6″), 145.8, 145.8 (C-7′, 7″), 114.6, 114.9 (C-8′, 8″), 166.5, 165.7 (C-9′, 9″). Compared with reported data [18], compound **B** was identified as 1,5-*O*-dicaffeoylquinic acid. 

Compound **C** EIS-MS, *m/z* 369.1518 [M + H]^+^, 367.1044 [M − H]^−^; ^1^H-NMR (400 MHz, DMSO-*d*_6_) *δ* (ppm): 2.12 (2H, m, H-2e, 6a), 1.77 (1H, dd, *J* = 9.6, 12.4 Hz, H-2a), 5.02 (1H, d, *J* = 3.6 Hz, H-3), 3.57 (1H, m, H-4), 3.88 (1H, d, *J* = 8.8 Hz, H-5), 1.93 (1H, dd, *J* = 13.6, 3.2 Hz, H-6e), 7.03 (1H, s, H-2′), 6.78 (1H, d, *J* = 8.0 Hz, H-5′), 6.98 (1H, d, *J* = 8.0 Hz, H-6′), 7.39 (1H, d, *J* = 16.0 Hz, H-7′), 6.11 (1H, d, *J* = 16.0 Hz, H-8′), 3.58 (3H, s, -OCH_3_); ^13^C-NMR (100 MHz, DMSO-*d*_6_) *δ* (ppm): 73. 5(C-1), 35. 6 (C-2), 71. 5 (C-3), 69.8 (C-4), 67.3 (C-5), 37. 7(C-6), 125. 8 (C-1′), 115. 1 (C-2′), 145. 6 (C-3′), 148.9 (C-4′), 116. 3 (C-5′), 121. 8 (C-6′), 146. 08 (C-7′), 114. 3 (C-8′), 165. 8 (C-9′), 52. 3 (-OCH_3_), 174. 1 (C-7). Compared with reported data [19], compound **C** was identified as 3-*O*-Caffeoylquinic acid methyl ester.

Compound **D** ESI-MS, *m/z* 517.1360 [M + H]^+^, 515.1258 [M − H]^−^; ^1^H-NMR (DMSO-*d_6_*, 400 MHz) *δ* (ppm): 1.91 (1H, dd, *J* = 9.2, 13.2 Hz, H-2), 2.32 (1H, dd, *J* = 10.4, 13.2 Hz, H-2), 5.23 (1H, m, H-3), 3.62 (1H, d, *J* = 6.0 Hz, H-4), 4.08 (1H, br s, H-5), 2.28 (2H, br s, H-6), 7.05 (1H, br s, H-2′), 6.78 (1H, d, *J* = 8.0 Hz, H-5′), 7.00 (1H, d, *J* = 8.0 Hz, H-6′), 7.48 (1H, d, *J* = 16.0 Hz, H-7′), 6. 21 (1H, d, *J* =16.0 Hz, H-8′), 7. 05 (1H, br s, H-2″), 6. 78 (1H, d, *J* = 7.2 Hz, H-5″), 7.00 (1H, d, *J* = 8.0 Hz, H-6″), 7. 47 (1H, d, *J* = 15.8 Hz, H-7″), 6. 21 (1H, d, *J* = 16.0 Hz, H-8″); ^13^ C-NMR (DMSO-*d_6_*, 100 MHz) *δ* (ppm): 79.9 (C-1), 36.3 (C-2), 70.5 (C-3), 71.4 (C-4), 68.0 (C-5), 34.6 (C-6), 126.0 (C-1′), 115.3 (C-2′), 146.1 (C-3′), 148.9 (C-4′), 116.3 (C-5′), 121.8 (C-6′), 145.8 (C-7′), 114.9 (C-8′), 166.5 (C-9′), 126.0 (C-1″), 115.3 (C-2″), 146.1 (C-3″), 148.9 (C-4″), 116.3 (C-5″), 121.7 (C-6″), 145.6 (C-7″), 114.7 (C-8″), 165.7 (C-9″), 173.1 (-COOH). Compared with reported data [20], compound **D** was identified as 1, 3-*O*-dicaffeoylquinic acid.

Compound **E** ESI-MS, *m/z* 633.1460 [M + H]^+^, 631.1320 [M − H]^−^; ^1^H-NMR (DMSO-*d*_6_, 400 MHz) *δ* (ppm): 2.41–2.67 (2H, m, overlap, H-2), 5.30 (1H, m, H-3), 3.86 (1H, dd, *J* = 3.2, 8.4Hz, H-4), 5.19 (1H, m, H-5), 1.98 (1H, m, H-6), 2.47 (1H, m, H-6, overlap), 4.27 (1H, m, H-2′), 2.41–2.67 (2H, m, H-3′), 7.01, 7.06 (each 1H, br s, H-2′, 2″), 6.78 (each 1H, d, *J* = 8.0 Hz, H-5′, 5″), 7.01 (each 1H, d, *J* = 8.0 Hz, H-6′, 6″), 7.50, 7.50 (each 1H, d, *J* = 15.6 Hz, H-7′, 7″), 6.27, 6.23 (each 1H, d, *J* = 15.6 Hz, H-8′, 8″); ^13^C-NMR (DMSO-*d*_6_, 100 MHz) *δ* (ppm): 79.2 (C-1), 32.3 (C-2), 71.6 (C-3), 69.0 (C-4), 70.2 (C-5), 35.8 (C-6), 172.4 (C-7), 174.9 (C-1′), 67.2 (C-2′), 39.7 (C-3′, overlap), 170.2 (C-4′), 125.9, 126.0 (C-1″, 1′′′), 115.3, 115.5 (C-2″, 2′′′), 145.9, 146.5 (C-3″, 3′′′), 148.9, 149.0 (C-4″, 4′′′), 116.3, 116.3 (C-5″, 5′′′), 121.8, 121.9 (C-6″, 6′′′), 146.1, 146.1 (C-7″, 7′′′), 114.2, 114.4 (C-8″, 8′′′), 165.7, 166.4 (C-9″, 9′′′). Compared with reported data [21], compound **E** was identified as 1,5-*O*-dicaffeoyl-3-*O*-(4-maloyl)-quinic acid.

Compound **F** ESI-MS, *m/z* 517.1334 [M + H]^+^, 515.1212 [M − H]^−^; ^1^H-NMR (CD_3_OD, 400 MHz) *δ* (ppm): 2.09–2.32 (4H, m, H-2, 6), 4.38 (1H, m, H-3), 5.13 (1H, dd, *J* = 3.2, 9.2 Hz, H-4), 5.62 (1H, m, H-5), 7.01, 7.03 (each 1H, H-2′, 2″), 6.74, 6.76 (each 1H, d, *J* = 8.0 Hz, H-5′, 5″), 6.90, 6.92 (each 1H, dd, *J* = 2.4, 8.0 Hz, H-6′, 6″), 6.19, 6.29 (each 1H, d, *J* = 16.0 Hz, H-8′, 8″), 7.52, 7.60 (each 1H, d, *J* = 16.0 Hz). ^13^C-NMR (CD_3_OD, 100 MHz) *δ* (ppm): 76.1 (C-1), 38.4 (C-2), 69.4 (C-3), 75.8 (C-4), 69.0 (C-5), 39.4 (C-6), 176.9 (C-7), 127.6, 127.7 (C-1′, 1″), 115.2, 115.2 (C-2′, 2″), 146.8 (C-3′, 3″), 149.7 (C-4′, 4″), 116.5 (C-5′, 5″), 123.1, 123.1 (C-6′, 6″), 147.6, 147.7 (C-7′, 7″), 114.7, 114.8 (C-8′, 8″), 168.3, 168.6 (C-9′, 9″). Compared with reported data [22], compound **F** was identified as 4, 5-*O*-dicaffeoyl-quinic acid.

Compound **G** ESI-MS, *m/z* 617.1516 [M + H]^+^, 615.1369 [M − H]^−^; ^1^H-NMR (DMSO-*d_6_*, 400 MHz) *δ* (ppm): 2.39–2.55 (2H, m, H-2, overlap), 5. 25 (1H, m, H-3), 3. 84 (1H, d, *J* = 8.8 Hz, H-4 ), 5.22 (1H, m, H-5 ), 1.92 (1H, m, H-6), 2.39–2.54 (1H, m, H-6, overlap), 2.39–2.54 (4H, m, H-2′, H-3′, overlap), 7.07, 7.07 (each 1H, br s, H-2″, 2′′′), 6.78, 6.78 (each 1H, d, *J* = 8.0 Hz, H-5″, 5′′′), 7.01, 7.01 (each 1H, d, *J* = 8.0 Hz, H-6″, 6′′′) 7.49, 7.50 (each 1H, d, *J* = 16.0 Hz, H-7″, 7′′′), 6.25, 6.27 (each 1H, d, *J* = 16.0 Hz, H-8″, 8′′′). ^13^C-NMR (DMSO-*d_6_*, 100 MHz) *δ* (ppm): 80.0 (C-1), 32.3 (C-2), 70.3 (C-3), 71.9 (C-4), 69.6 (C-5), 36.6 (C-6), 173.7 (C-7), 172.7 (C-1′), 29.4 (C-2′), 29.2 (C-3′), 172.1 (C-4′), 125.9, 126.0 (C-1″, 1′′′), 115.4, 115.4 (C-2″, 2′′′), 146.1, 146.1 (C-3″, 3′′′), 149.0, 149.0 (C-4″;, 4′′′), 116.3, 116.4 (C-5″, 5′′′), 122.6, 122.7 (C-6″, 6′′′), 145.8, 145.8 (C-7″, 7′′′), 114.5, 114.6 (C-8″, 8′′′), 166.5, 165.6 (C-9″, 9′′′). Compared with reported data [2], compound **G** was identified as 1,5-*O*-dicaffeoyl-3-*O*-succinylquinic acid.

Compound **H** ESI-MS, *m/z* 617.1529 [M + H]^+^, 615.1374 [M − H]^−^; ^1^H-NMR (DMSO-*d_6_*, 400 MHz) *δ* (ppm): 2.32–2.60 (2H, m, overlap), 4.24 (1H, m, H-3), 4.92 (1H, m), 5.46 (1H, m), 2.02–2.07, 2.32–2.60 (1H, m, H-6, overlap), 2.32–2.60 (4H, m, H-2′, 3′), 7.06, 7.06 (each 1H, br s, H-2″, 2′′′), 6.77, 6.79 (each 1H, d, *J* = 8.0 Hz, H-5″, 5′′′), 7.01, 7.01 (each 1H, d, *J* = 8. 0 Hz, H-6″, 6′′′), 7.47, 7.48 (each1H, d, *J* = 15.6 Hz, H-7″, 7′′′), 6.20, 6.24 (each 1H, d, *J* = 15.6 Hz, H-8″, 8′′′); ^13^C-NMR (DMSO-*d_6_*, 100 MHz) *δ* (ppm): 79.5 (C-1), 34.7 (C-2), 65.6 (C-3), 74.4 (C-4), 67.2 (C-5), 36.8 (C-6), 173.8 (C-7), 172.2 (C-1′), 29.4 (C-2′), 29.2 (C-3′), 172.1 (C-4′), 126.0, 126.0 (C-1″, 1′′′), 115.3, 115.4 (C-2″, 2′′′), 146.1, 146.1 (C-3″, 3′′′), 148.9, 149.0 (C-4″, 4′′′), 116.3, 116.4 (C-5″, 5′′′), 121.7, 121.9 (C-6″, 6′′′), 146.1, 146.1 (C-7″, 7′′′), 114.0, 114.8 (C-8″, 8′′′), 165.7, 166.2 (C-9″, 9′′′). Compared with reported data [2], compound **H** was identified as 1-,5-*O*-dicaffeoyl-4-*O*-succinylquinic acid.

#### 2.2.2. Identification of New Compounds

**Figure 3:**
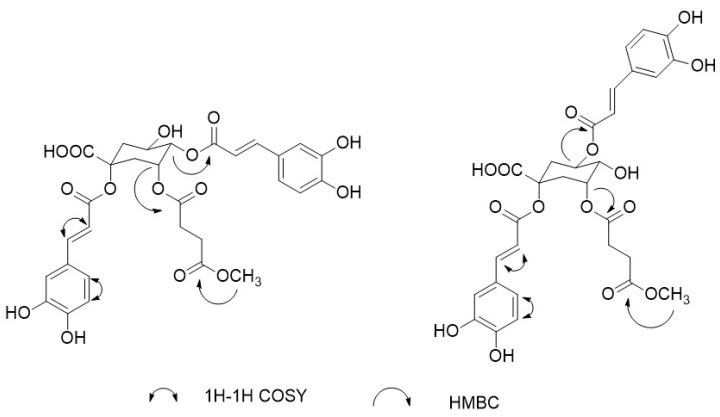
Key HMBC and ^1^H-^1^H COSY correlations of new compounds.

Compound **I** was isolated as a white amorphous powder. HR-ESI-MS spectrum gave a molecular ion peak at *m/z* 631.16532 [M + H]^+^ (calc.631.16575), indicating a molecular formula of C_30_H_30_O_15_. ^1^H-NMR and ^13^C-NMR spectra exhibited four doublets with coupling constants of 15.6 Hz characteristic for trans olefinic protons (*δ*_H_ 6.23, 6.31, 7.47, 7.50). The coupling pattern of six aromatic proton signals (7.06, 7.07 (each 1H, br s), 6.66, 6.78 (each 1H, d, 8.4 Hz), 7.00, 7.03 (each 1H, d, 8.4 Hz)) appearing as two ABX systems in ^1^H-^1^H COSY (Figure 3) spectrum, which indicated the presence two caffeic acid moieties [22]. The ^1^H-NMR signals at *δ*_H_ 2.31–2.47 (4H, m) and ^13^C-NMR signals at *δ*_C_ 28.8, 29.3, 172.4, 172.6 indicated the presence of succinyl moiety. The ^1^H-NMR signals at *δ*_H_ 3.48 (3H, s) and ^13^C-NMR signals at *δ*_C_ 51.8 indicated the presence of methoxyl moiety. The rest of the signals of ^1^H-NMR and ^13^C-NMR were attributed to a quinic acid moiety. The low-filed signals of *δ*_H_ 5.38 (1H, m, H-3), and *δ*_H_ 4.88 (1H, dd, 3.2 Hz, 8.8 Hz) indicated the substituted of 3-OH and 4-OH, the low-filed of *δ*_C_ 79.7 indicated the substituted of 1-OH. Therefore, compound **I** was a caffeoyl quinic acid derivatives with three acyl groups at 1, 3, and 5 positions of quinic acid. 

The location of the two caffeoyl groups and one succinyl group on the quinic acid moiety was deduced by HMBC spectrum (Figure 3). The correlation of *δ*_H_ 5.38 (1H, m, H-3) and *δ*_H_ 4.88 (1H, dd, 3.2 Hz, 8.8 Hz) to 171.4, 166.5, separately, which indicated the substitution of a caffeoyl group on C-4, a succinyl group on C-3. Moreover, the correlation of *δ*_H_ 3.48 (3H, s) to *δ*_C_ 172.4 indicated the esterification of succinic acid. Conclusively, the other caffeoyl group was unambiguously connected to 1-OH. Therefore, the compound was identified as 1,4-*O*-dicaffeoyl-3-succinyl methyl ester quinic acid. The detail ^1^H-NMR and ^13^C-NMR data were listed in Table 2. 

Compound **J** was isolated as a white amorphous powder. HR-ESI-MS spectrum gave a molecular ion peak at *m/z* 631.16814 [M + H]^+^ (calc.631.16575), indicating a molecular formula of C_30_H_30_O_15_.The molecular ion peak was at *m/z* 631.1640 [M + H]^+^. ^1^H-NMR and ^13^C-NMR spectra were similar with compound **I** and also exhibited two caffeic acid moieties, one succinyl moiety, and one -OCH_3_ (Table 2), which indicated that compound **J** was also a caffeoylquinic acid derivatives with three acyl groups. The low-filed signals of *δ*_H_ 5.26 (1H, m, H-3), and *δ*_H_ 5.23 (1H, m) indicated the substituted of 3-OH and 5-OH, and the low-filed of *δ*_C_ 79.7 indicated the substituted of 1-OH. These data were similar with 1,5-*O*-dicaffeoyl-3-*O*-succinylquinic acid as reported [22], and the differences were the presence of one -OCH_3_.

The location of the -OCH_3_ was deduced by HMBC spectrum (Figure 3). The correlation of *δ*_H_ 3.51 (3H, s) and *δ*_C_ 171.7 indicated-OCH_3_ was located in succiny quinic acid. Therefore, compound **J** was identified as 1,5-*O*-dicaffeoyl-3-*O*-succinyl methyl ester quinic acid.

## 3. Experimental

### 3.1. Material and Reagents

The solvents used in this experiment, including petroleum ether (Pet, 60–90 °C), ethyl acetate (EtOAc), methanol (MeOH), and 95% ethanol were all of analytical grade (Tianjin Fuyu Fine Chemical Co., Ltd, Tianjin, China). Methanol and acetonitrile used for HPLC analysis were of chromatographic grade (Oceanpak Alexative Chemical, Ltd, Gothenburg, Sweden). The purified water was prepared by an osmosis Milli-Q system (Millipore, Bedford, MA, USA). Burdock root was purchased from Yiwei Food Co. Ltd. (Pei Town, Jiangsu Province, China) and identified as the roots of *Arctium lappa* L. by Xiao Wang (Shandong analysis and Test Center, Qilu University of Technology, Shandong Academy of Sciences, Jinan, China). The voucher specimen (No. sdatc-2017-023) was deposited at Shandong analysis and test center.

### 3.2. Apparatus

HSCCC separation was carried out using a TBE-300C instrument (Tauto Biotech, Shanghai, China) with 300 mL of multilayer coil (I.D. 1.9 mm) and a 20 mL sample loop. The revolution speed was regulated in the range of 0–1000 rpm. The HSCCC system was equipped with a DC-0506 constant temperature circulating device (Tauto Biotech, Shanghai, China) to stabilize the separation temperature. A TBP-5002 constant flow pump was used to pump the solvents. Continuous monitoring of the effluent was achieved using an 8823B-UV detector (Beijing BINTA Instrument Technology Co., Ltd., Beijing, China) at 280 nm. A Model 3057-11 portable recorder (Chongqing Sichuan Instrument Automation Co., Ltd. Chongqing, China) was used to record the chromatograms.

The analysis of all samples was performed using a Waters Alliance system including a Waters 2998 Photodiode Array Detection system, a Waters 2695 Multi-Solvent delivery system, a Waters 2695 system controller, a Waters 2695 pump, and an Empower 3 Workstation (Waters, Milford, MA, USA).

### 3.3. Pre-Processing of Crude Sample

Powdered burdock roots (1.0 kg) was extracted three times with 10 L of 95% ethanol under reflux (2 h for each). After filtration, the solvent was concentrated in vacuo at 45 °C to afford a residue (83 g). The residue was dissolved in 1000 mL of water and partitioned with equal volumes of Pet and EtOAc in a separator funnel (each for three times) successively. The EtOAc layers were combined and evaporated under reduced pressure at 45 °C giving 11.12 g of EtOAc extract. The EtOAc extract was further separated by MCI column chromatography (4 × 40 cm) and eluted with 10% (0.9 L), 40% (2 L), 70% (1.65 L), and 100% (0.55 L) methanol, and 40% elution solvent was fractionated into Fr. 1 (1.12 g), Fr. 2 (3.61 g) and Fr. 3 (0.8 g) according to HPLC analysis (Figure S1).

### 3.4. Optimization of HPLC Conditions

The ethyl acetate extract of burdock roots was analyzed by HPLC. The mobile phases including methanol/acetonitrile-water containing 0.1% HCOOH and column including Phenomenex Gemini-NX C18 (250 mm × 4.6 mm, 5µm), YMC-Pack ODS-A (250 mm × 4.6 mm, 5 µm), Waters Symmetry C18 (250 mm × 4.6 mm, 5µm), Spax Technologies Inc. Amethyst C18-H (250 mm × 4.6 mm, 5µm) were tested for separation. The results showed that the baseline separation of the target compounds were obtained when the mobile phase was acetonitrile (**A**)-water containing 0.1% HCOOH (**B**) (0–7 min, 15% **A**; 7–8 min, 15–20% **A**; 8–29 min, 20% **A**; 29–30 min, 20–25% **A**; 30–50 min, 25–30% **A**), and the column was Phenomenex Gemini-NX C18 (250 mm × 4.6mm, 5µm, 110 Å). The flow-rate was 1.0ml/min, and the effluent was monitored at 280 nm by a DAD detector.

The HPLC chromatography of crude extract and fractionated samples (Fr. 1–3) by MCI macroporous resin is shown in Figure 2, which revealed that Fr. 1 mainly contains compounds **A**–**C**, Fr. 2 mainly contains compound **D**, and Fr. 3 mainly contains compounds **E**–**J**.

### 3.5. Selection and Preparation of the Two-Phase Solvent System

The selection of a two-phase solvent system with suitable partition coefficient (*K*_D_) was very important in the entire work of HSCCC separation. The *K*_D_ value was measured as follows: about 2.0 mg of crude sample was added to a test tube, to which 2 mL of each-phase solvent were added. The test tube was shaken vigorously to promote the dissolve of the samples. After the equilibration was established, both the upper phase and lower phase were analyzed by HPLC. The *K*_D_ value was defined as the peak area of target compounds in the stationary phase divided by that in the mobile phase [16].

The designate solvent was poured into a separatory funnel and shaken rapidly. After thoroughly equilibrated at room temperature, the two phases were separated for HSCCC separation.

### 3.6. Separation Procedure

For each separation, the separation column was first filled with the upper phase (stationary phase) at a flow rate of 20.0 mL/min. Then, the apparatus was rotated forward at 800 rpm, while the lower phase was pumped through the column as mobile phase at a flow-rate of 5.0 mL/min. After the hydrodynamic equilibrium system was established, the sample solution (120 mg of sample dissolved in 4 mL of lower phase and 4 mL of upper phase) was injected via the sample port. The separation process was kept at 25 °C. The effluents were continuously monitored with a UV detector at 280 nm and collected manually according to the profile of HSCCC chromatography.

### 3.7. Semi-Preparative HPLC Separations

Semi-preparative HPLC separations were operated at YMC C18 (10.0 mm × 250 mm, 5μm) column using the solvent composed of acetonitrile-water containing 0.1% HCOOH (25:75, *v*/*v*) and methanol-water containing 0.1% HCOOH (25:75, *v*/*v*) at a flow-rate of 3.0 mL/min and monitored at 280 nm. 

### 3.8. Identification of Compounds

The chemical structures of all compounds were determined by 1D, 2D-NMR spectrometry performed on a Bruker AV-400 spectrometer (Bruker BioSpin, Rheinstetten, Germany). HR-ESI-MS experiments were performed on an Agilent 6520 Q-TOF MS (Agilent, Santa Clara, CA, USA).

## 4. Conclusions

Caffeoylquinic acid derivatives are polyphenols that have abundant isomers with varieties of bioactivities. Due to the similarity of these compounds in chemical structure, repeat and multiple chromatography has often been adopted for their purification, which leads to the degrade and loss of these compounds. In this manuscript, a total of 10 caffeoylquinic acid derivatives including two new ones were obtained by one-step combination of HSCCC and semi-preparative HPLC. The results demonstrated that the combination of HSCCC with semi-preparative HPLC is a highly efficient means for preparative separation of the non-stable caffeoylquinic acid derivatives from natural products.

## Figures and Tables

**Table 1:** The *K*_D_ values of caffeoylquinic acid derivatives from burdock roots.

Sample	Solvent System (Pet-EtOAc-MeOH-H_2_O, *v*/*v*)	*K*_D_								
		A	B	C	D	E	F	G	H	I/J
Fr. 1	0:5:0:5	0.55	0.58	4.82						
	0:5:0.5:5	0.47	0.52	2.21						
Fr. 2	0:5:1:5				26.88					
	1:5:1:5				4.00					
	1:4:1:4				2.03					
Fr. 3	1:4:1:4					0.50	2.16	2.29	2.25	7.88
	1:4:2:3					0.19	0.29	0.39	0.41	0.76

**Table 2:** ^1^H-NMR (400 MHz) and ^13^C-NMR (100 MHz) data of compound **I** and **J** (DMSO-*d*_6_).

Position	Compound I		Compound J	
	*δ*_H_ (ppm, Mult, *J* in Hz)	*δ*_C_ (ppm)	*δ*_H_ (ppm, Mult, *J* in Hz)	*δ*_C_ (ppm)
1		79.7		78.8
2	2.31–2.47 (m, overlap)	32.3	2.34–2.58 (m, overlap)	31.4
3	5.38 (d, 3.6)	69.1	5.26 (m)	69.5
4	4.88 (dd, 3.2, 8.8)	74.7	3.85 (dd, 3.2, 8.8)	71.3
5	4.14 (m)	63.9	5.23 (m )	68.7
6	1.92 (m), 2.31–2.47 (m, overlap)	39.4 (overlap)	1.93 (m), 2.34–2.58 (m, overlap)	35.9
7		172.6		172.0
1′		171.4		171.7
2′	2.31–2.47 (m, overlap)	28.8	2.34–2.58 (m, overlap)	28.3
3′	2.31–2.47 (m, overlap)	29.3	2.34–2.58 (m, overlap)	28.8
4′		172.4		171.2
-OCH_3_	3.48 (s)	51.8	3.51 (s)	51.3
1″		126.0		125.3
2″	7.07 (1H, br s)	115.4	7. 07 (br s)	114.7
3″		145.6 (overlap)		145.8
4″		148.9		148.2
5″	6.78 (d, 8.4)	116.3	6.79 (d, 8.0)	115.7
6″	7.03 (d, 8.4)	121.9	7. 03 (dd, 8.0)	121.3
7″	7.50 (d, 15.6)	146.1	7.51 (d, 16.0)	145.2
8″	6.31 (d, 15.6)	114.5	6.25 (d, 16.0)	113.7
9″		166.5		165.1
1″′		125.9		125. 4
2′′′	7.06 (br s)	115.3	7. 07(br s)	114. 8
3′′′		145.6 (overlap)		145.8
4′′′		149.0		148.3
5′′′	6.77 (d, 8.4)	116.2	6.80 (d, 8.0)	115.7
6′′′	7.00 (d, 8.4)	121.8	7.05 (dd, 8.0)	121.3
7′′′	7.47 (d, 15.6)	146.1	7. 52 (d, 16.0)	145.3
8′′′	6.23 (d, 15.6)	114.5	6. 29 (d, 16.0)	114.0
9′′′		165.8		165.9

## Supplementary Materials

The following are available online. HPLC chromatograms of crude extract and purified compounds; NMR, MS spectrums of new compounds.
