# Supplementary material for: Semi-Preparative Separation of 10 Caffeoylquinic Acid Derivatives Using High Speed Counter-Current Chromatogaphy Combined with Semi-Preparative HPLC from the Roots of Burdock (Arctium lappa L.)
Source: Molecules. 2018 Feb 15;23(2):429. doi: 10.3390/molecules23020429 (PMC6017412; doi:10.3390/molecules23020429)
Supplement: Supplementary file 1 [file molecules-23-00429-s001.pdf]

Semi-Preparative Separation of 10 Caffeoylquinic Acid Derivatives Using High Speed Counter-Current Chromatography Combined with Semi-Preparative HPLC from the Roots of Burdock (*Arctium lappa* L.)

Zhenjia Zheng[1], XiaoWang[2], Pengli Liu[1], Meng Li[1], Hongjing Dong[2]*, Xuguang Qiao[1]*

[1] College of Food Science and Engineering, Shandong Agricultural University, Taian 271018, China; pengyou-jia@163.com (Z.Z.); milly.liu@163.com (P.L); Lemon982436@163.com (M.L.)

[2] Key Laboratory of TCM Quality Control Technology, Shandong Analysis and Test Center, Qilu University of Technology (Shandong Academy of Sciences), Jinan 250014, China; wangx@sdas.org (X.W.))

* Correspondence: donghongjing_2006@163.com (H.D.); xgqiao@sdau.edu.cn(X.Q.); Tel: +86-531-8260-5319 (H.D.); +86-538-8242-875 (X.Q.)

contents

Fig.S1 HPLC chromatograms of ethyl acetate extract and fractions from MCI 2
Fig.S2 HPLC chromatograms of purified compound of HSCCC from Fr.1 3
Fig.S3 HPLC chromatograms of purified compound of HSCCC from Fr.2 3
Fig.S4 HPLC chromatograms of purified compound of HSCCC from Fr.3 4
Fig.S5 MS spectrum of compound I 5
Fig.S6 ^{1}H-NMR of compound I 5
Fig.S7 ^{13}C-NMR of compound I 6
Fig.S8 HMBC of compound I 6
Fig.S9 HMQC of compound I 7
Fig.S10 ^{1}H-^{1}H COSY of compound I 7
Fig.S11 MS spectrum of compound J 8
Fig.S12 ^{1}H-NMR of compound J 8
Fig.S13 ^{13}C-NMR of compound J 9
Fig.S14 HMBC of compound J 9
Fig.S15 HMQC of compound J 10
Fig.S16 ^{1}H-^{1}H COSY of compound J 10

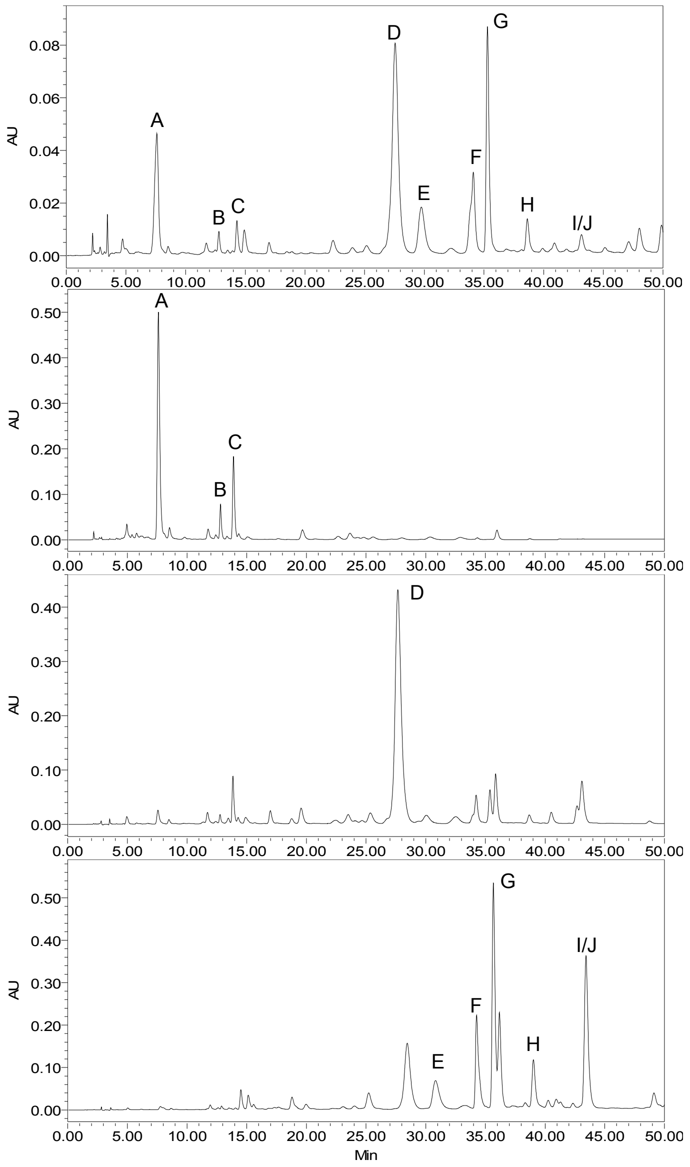

Fig. S1 HPLC chromatograms of ethyl acetate extract and fractions from MCI Conditions: column: Phenomenex Gemini-NX C18 (250 mm × 4.6 mm, 5μm, 110 Å); mobile phase: acetonitrile (A)-water containing 0.1% HCOOH (B) (0-7 min, 15%A; 7-8 min, 15%-20%A; 8-29 min, 20%A; 29-30 min, 20%-25%A; 30-50min, 25%-30%A), temperature: 25 ℃; flow-rate: 1.0 ml/min; UV monitor wavelength: 280 nm.

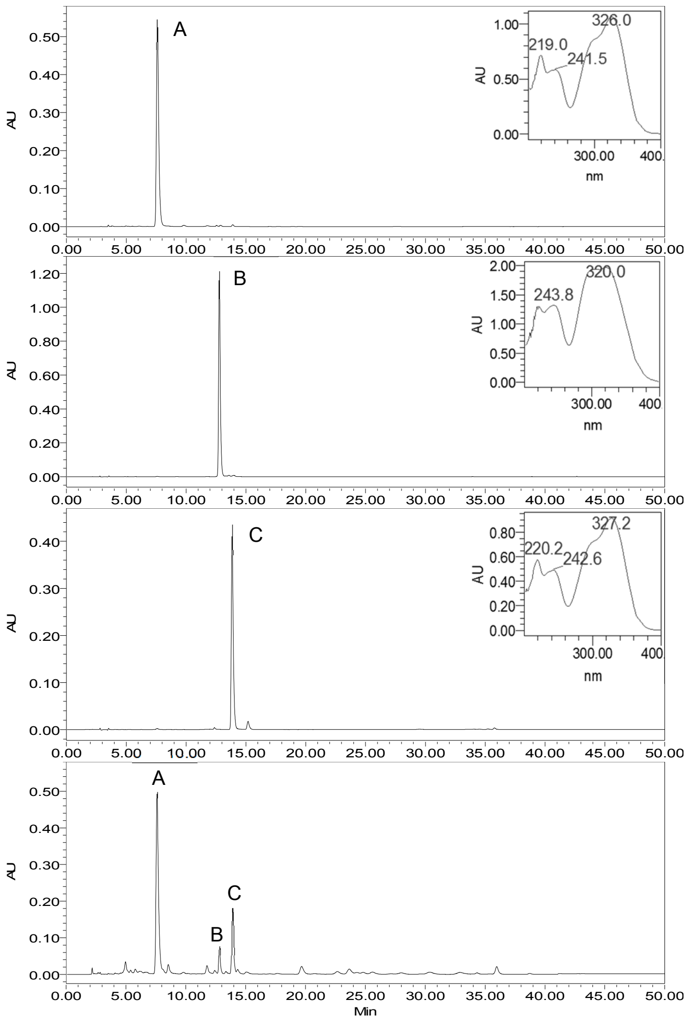

Fig. S2 HPLC chromatograms of purified compound of HSCCC from Fr.1

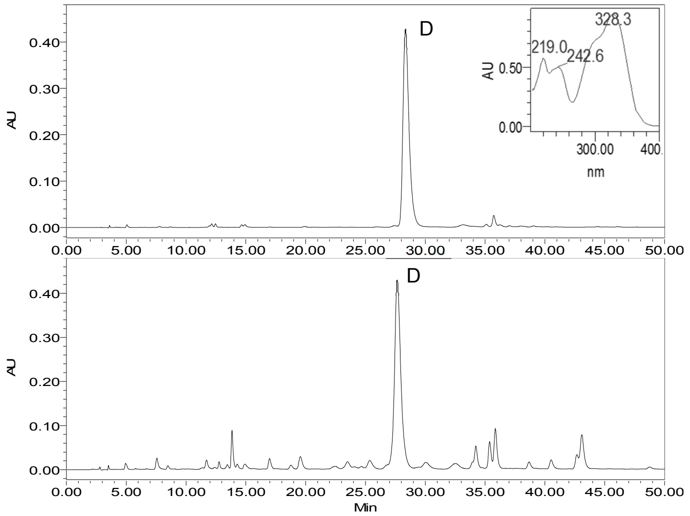

Fig. S3 HPLC chromatograms of purified compound of HSCCC from Fr.2

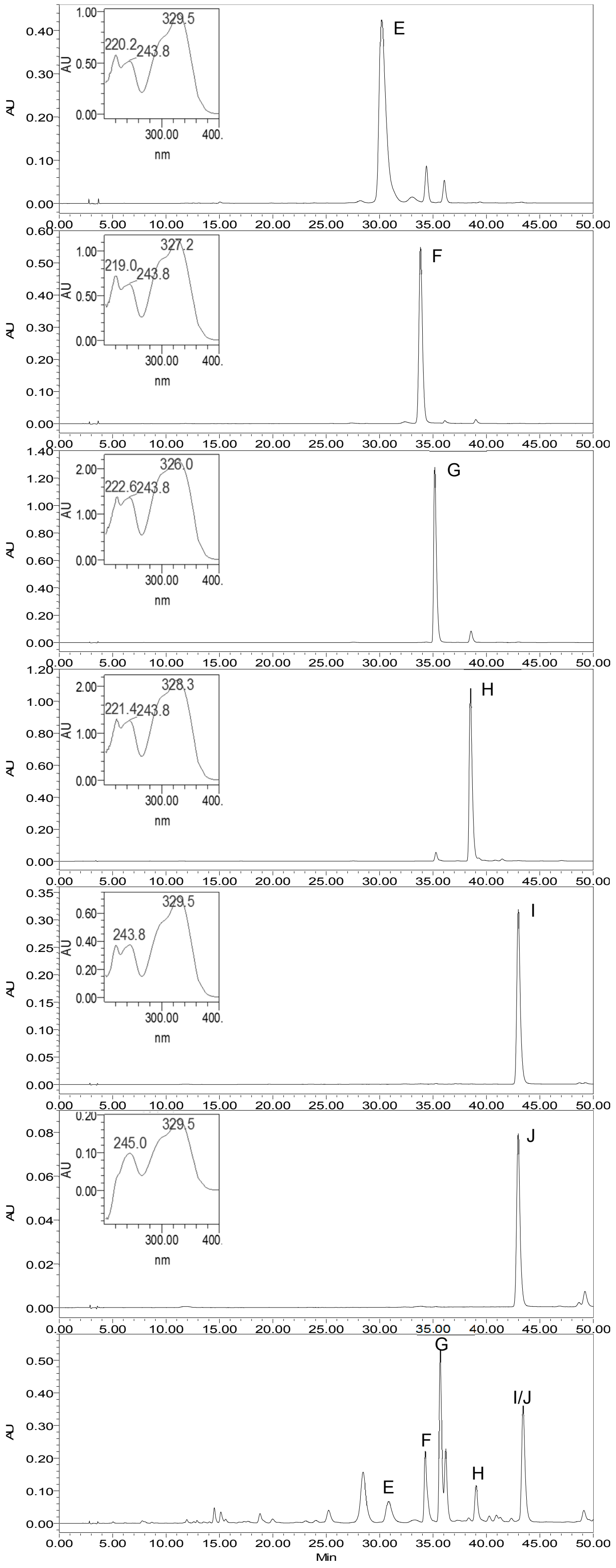

Fig. S4 HPLC chromatograms of purified compound of HSCCC from Fr.3

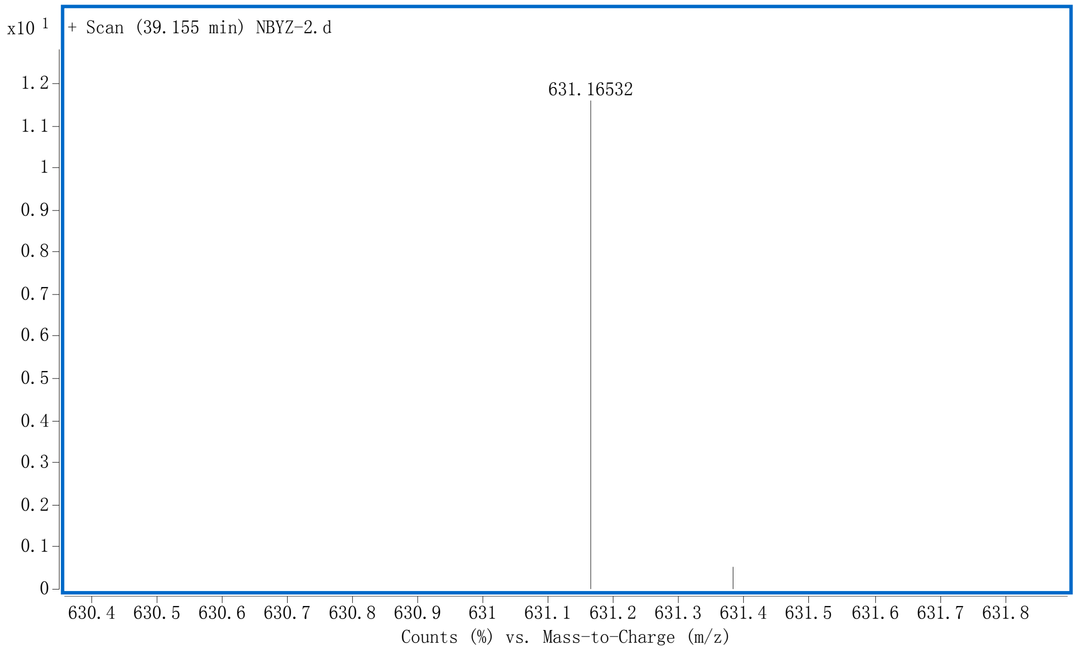

Fig. S5 MS spectrum of compound I

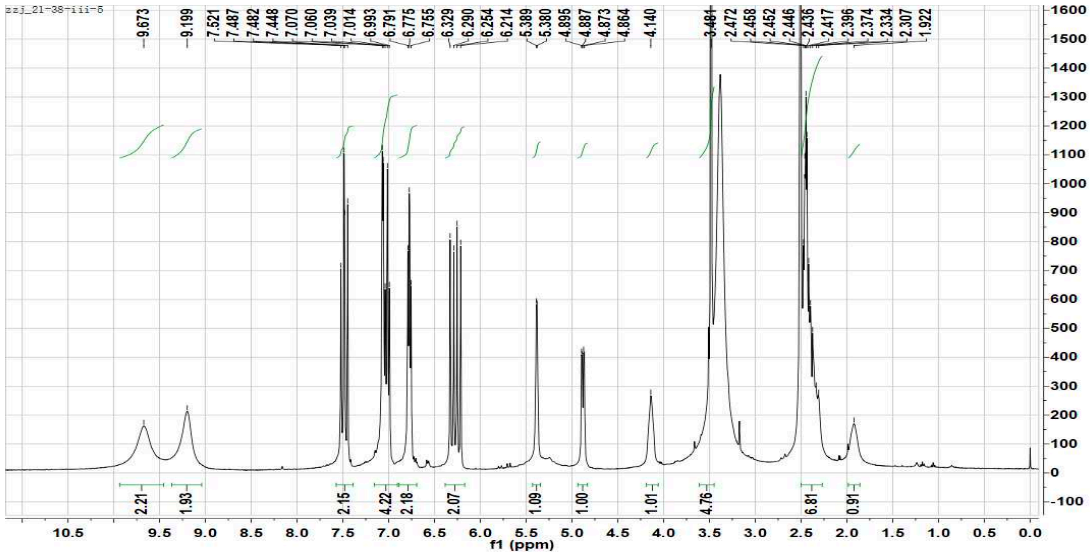

Fig. S6 ^{1}H-NMR of compound I

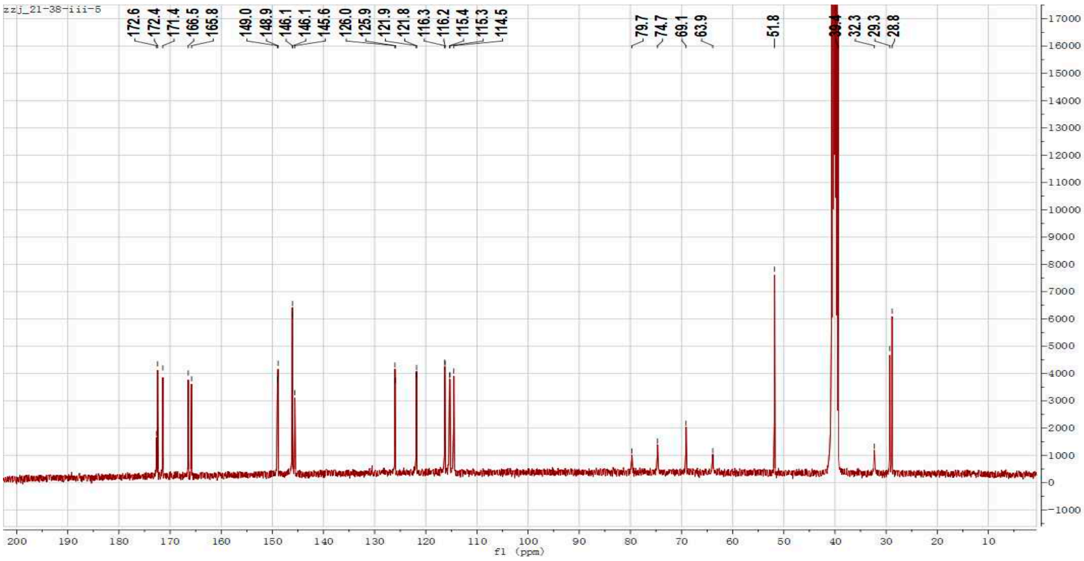

Fig. S7 ^{13}C-NMR of compound I

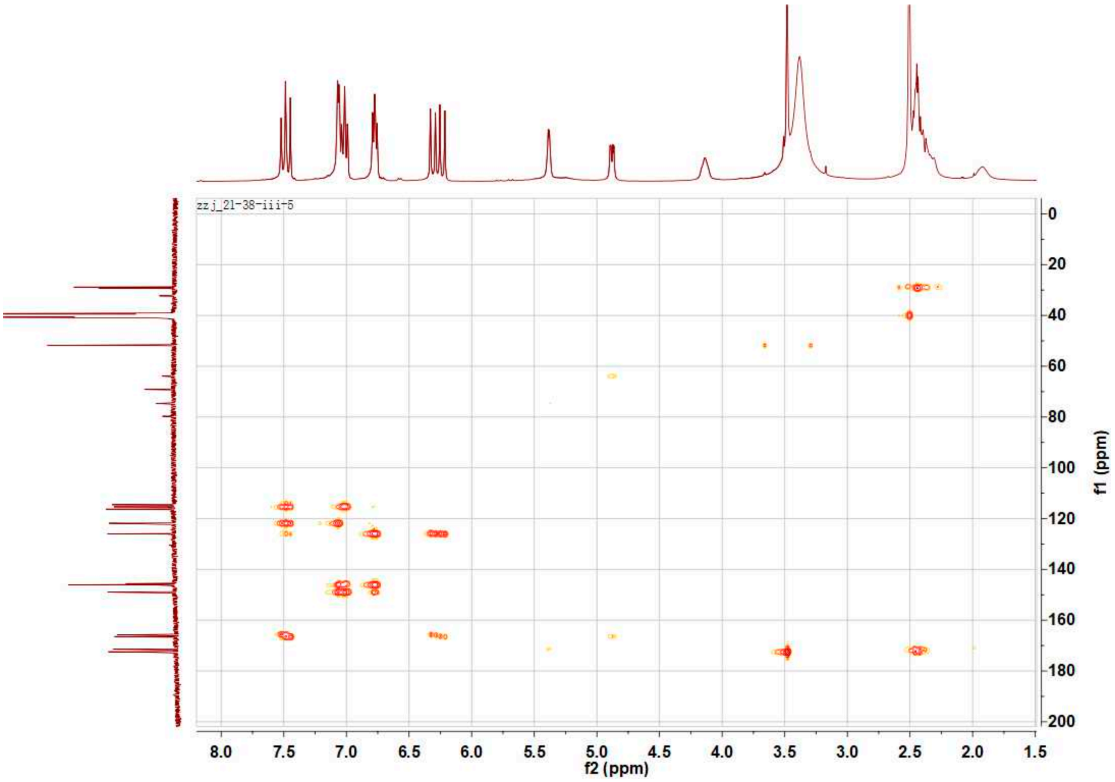

Fig. S8 HMBC of compound I

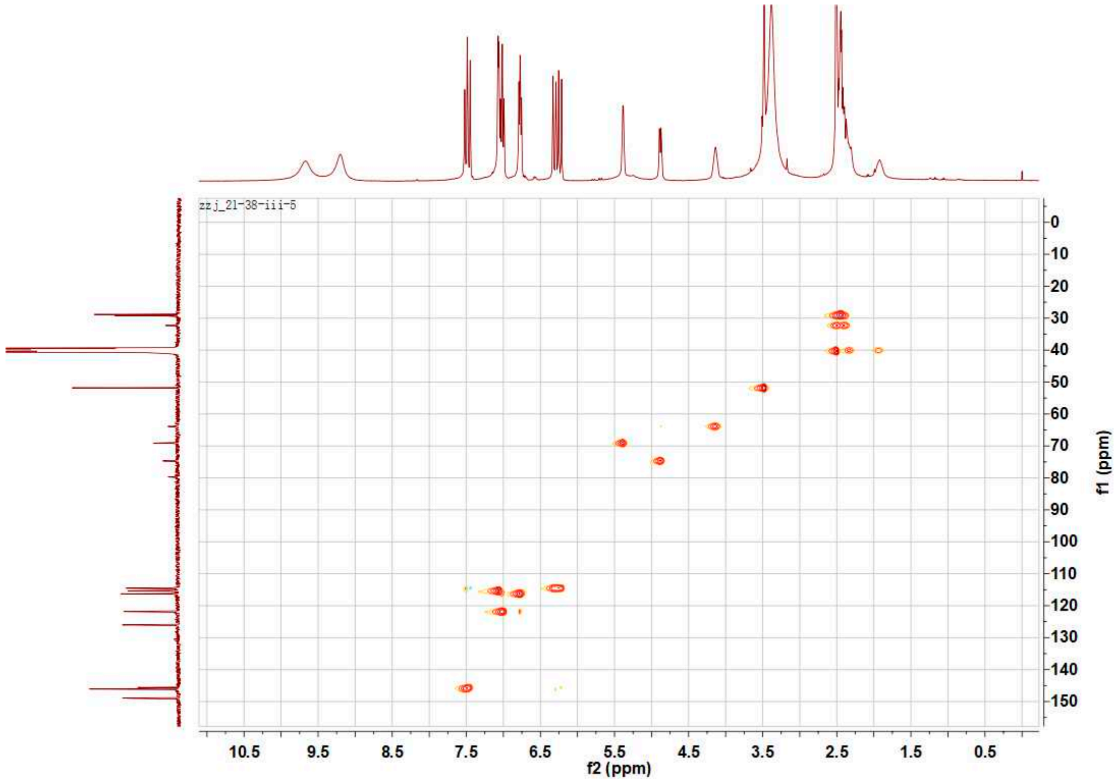

Fig. S9 HMQC of compound I

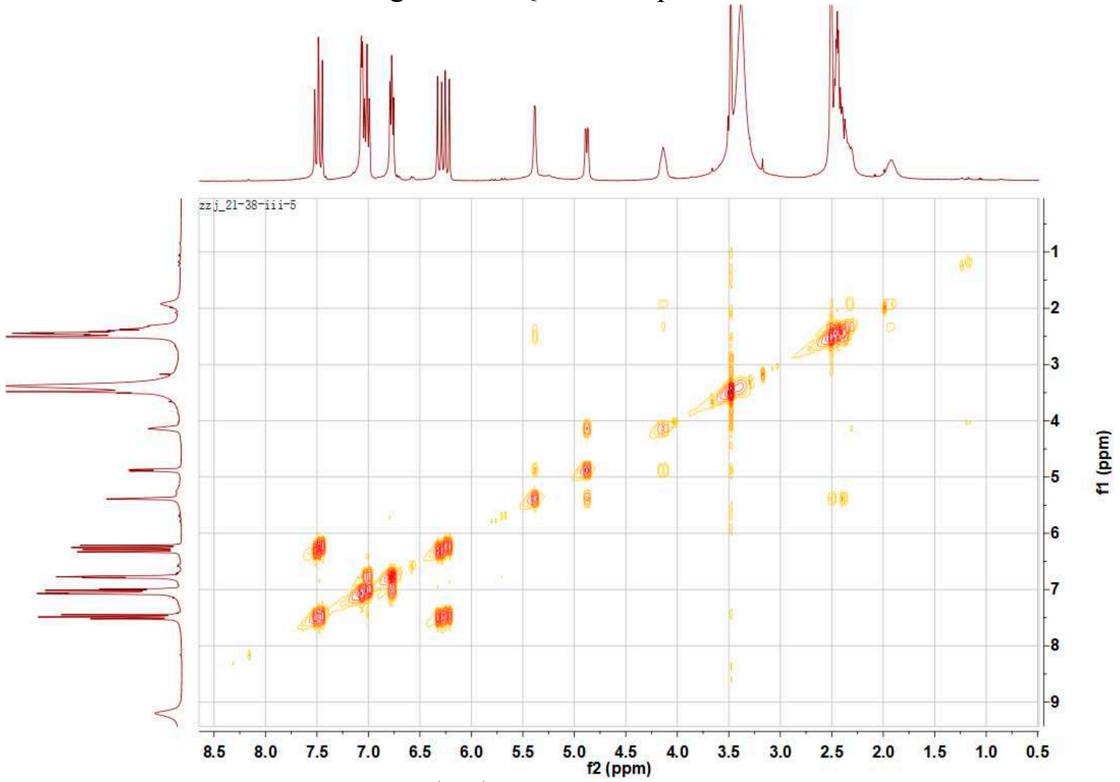

Fig. S10 ^{1}H-^{1}H COSY of compound I

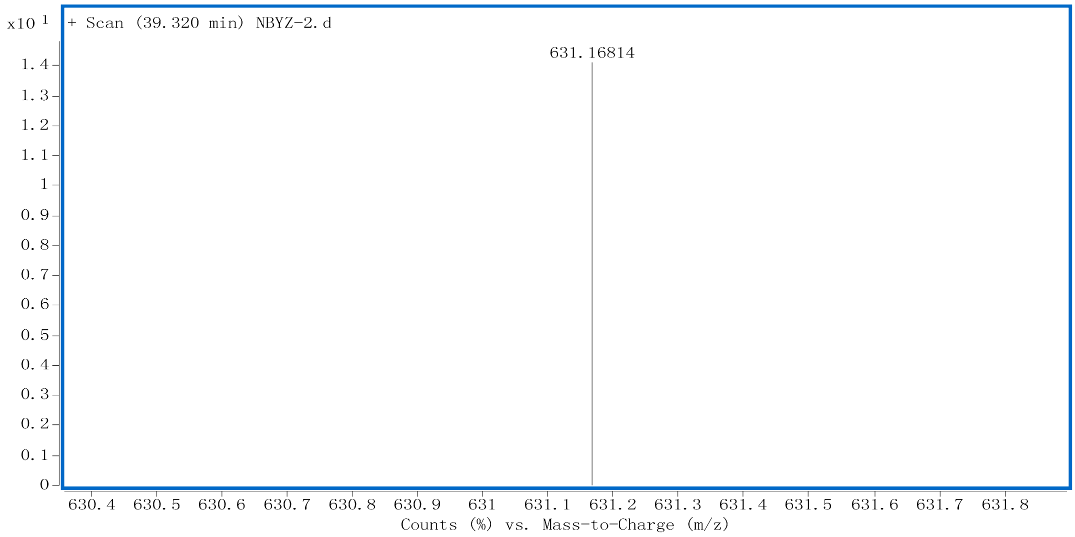

Fig. S11 MS spectrum of compound J

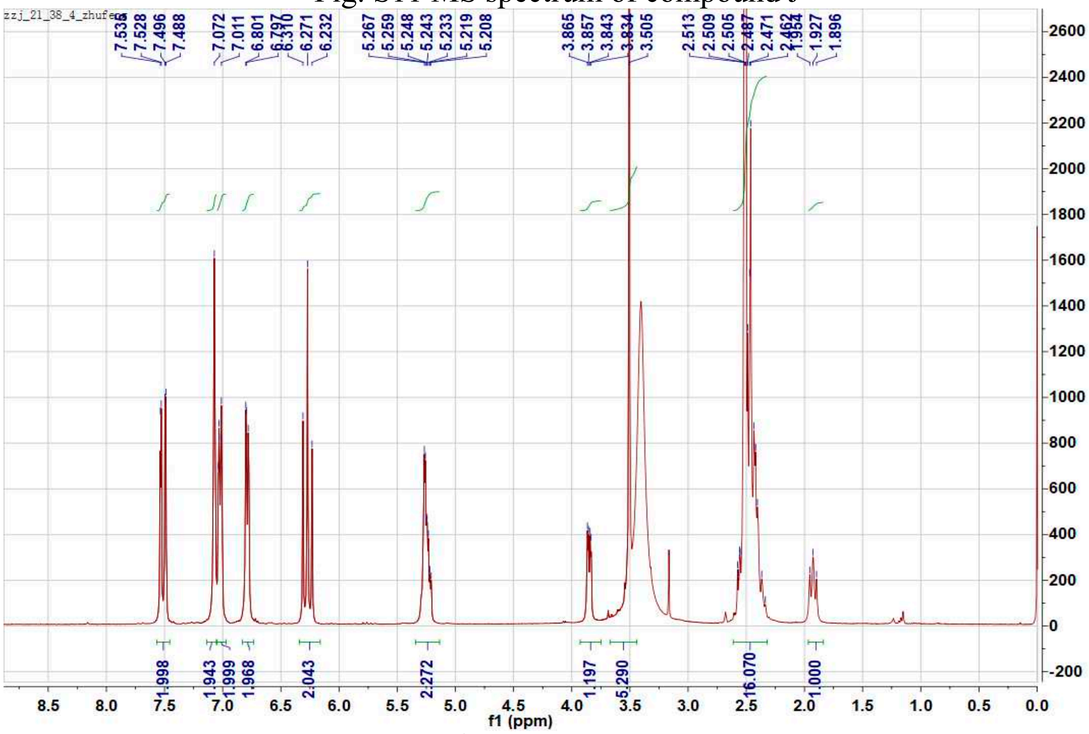

Fig. S12 ^{1}H-NMR of compound J

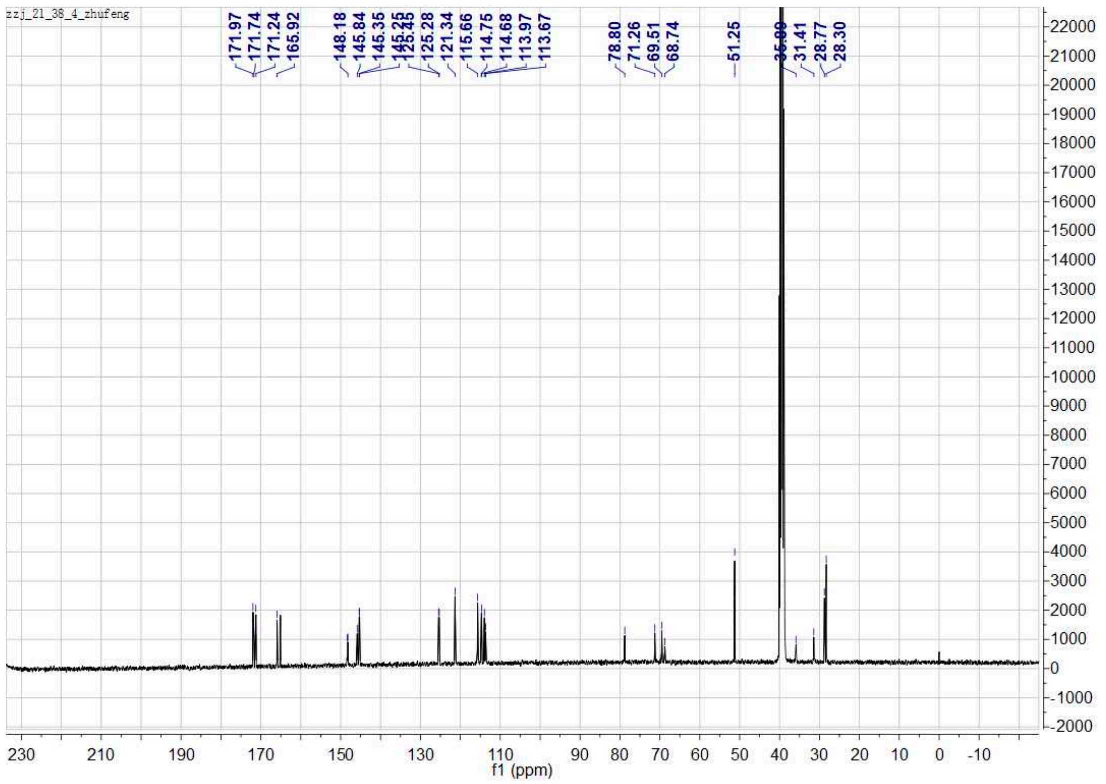

Fig. S13 ^{13}C-NMR of compound J

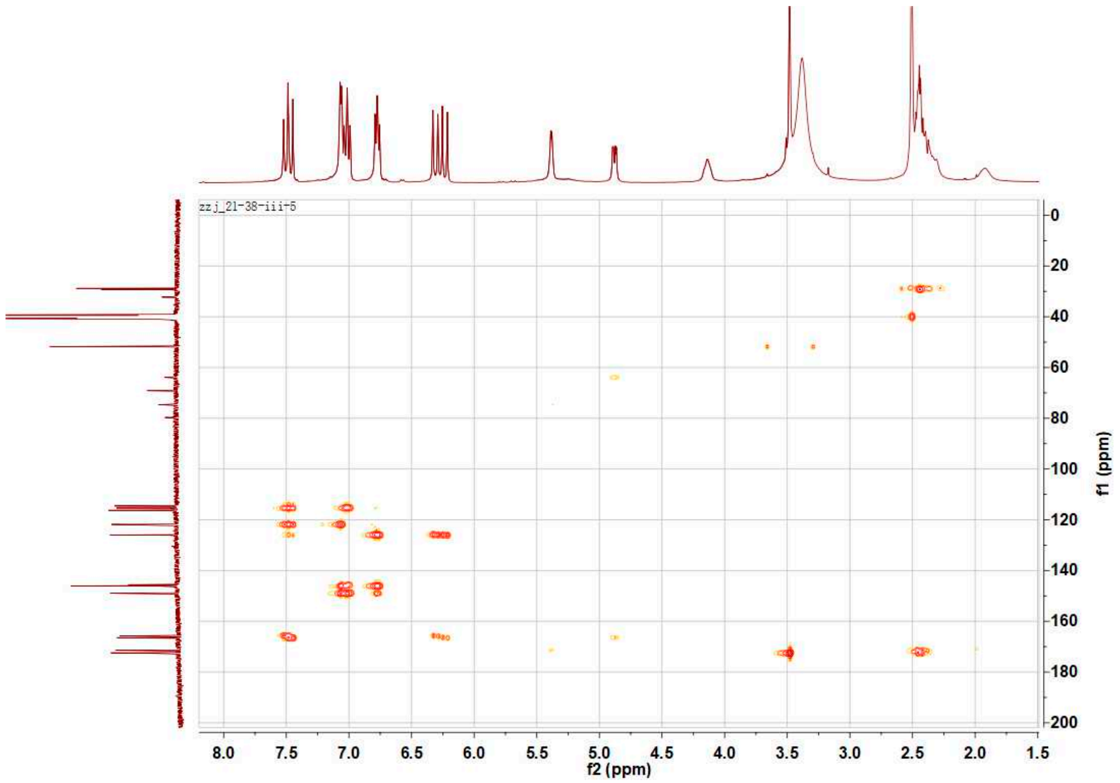

Fig. S14 HMBC of compound J

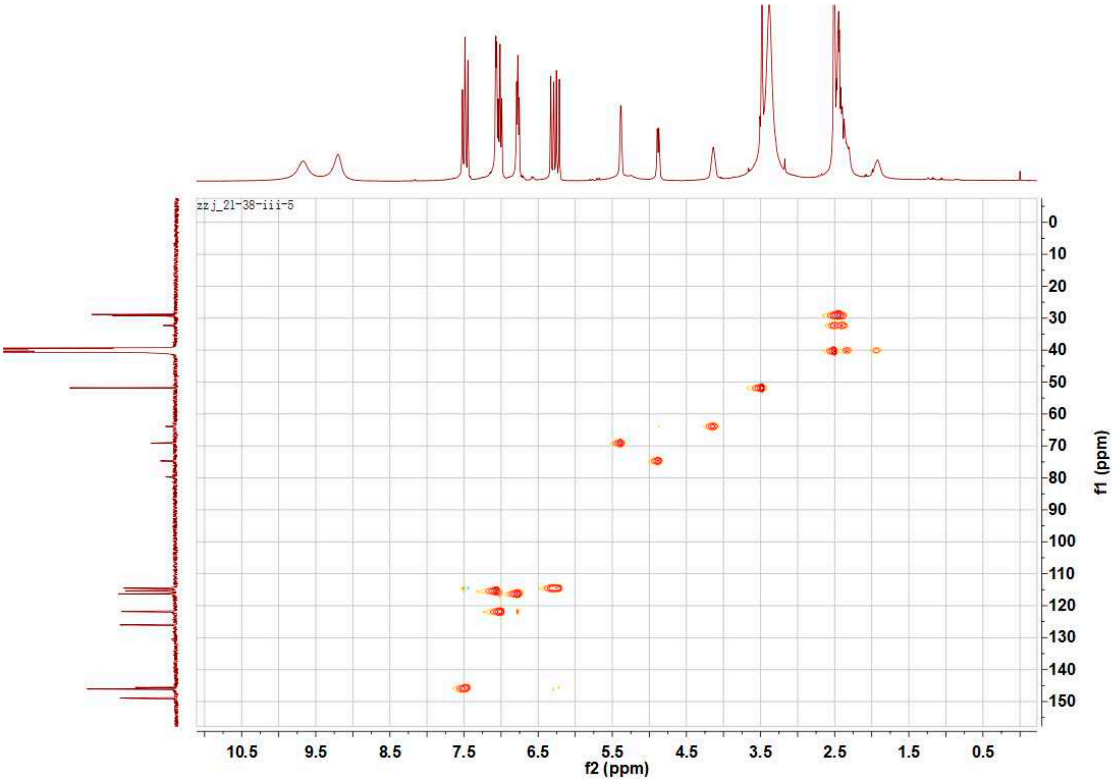

Fig. S15 HMQC of compound J

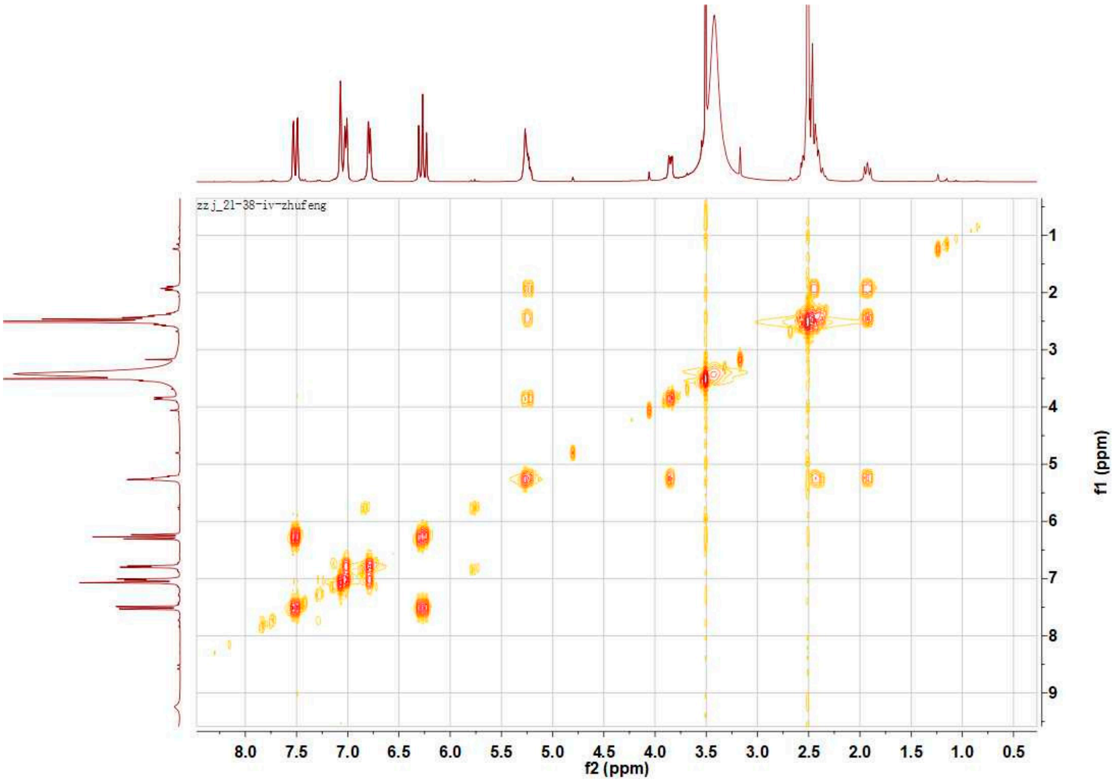

Fig. S16 ^{1}H-^{1}H COSY of compound J
